# Pocket Therapeutic Notes

**Published:** 1888-06

**Authors:** 


*Pocket Therapeutic Notes.* Pp. 157. Ferris & Co., Bristol. 1888.

This is an exceedingly handy and useful little work, containing a complete list of all new drugs and remedies, with full and clear descriptions of their therapeutic action. The alphabetic arrangement renders the finding of any remedy very easy; and the copious references to the leading authorities who have introduced or tried the various remedies assist further study where this is desirable. The labour which must have been expended in the preparation of these notes is scarcely suggested by the small size of the book; a peep inside the covers will show how much information can be given in very small space.

